# Intravenous N-Acetylcysteine in Patients With Dengue Fever With Marked Aminotransferase Elevation: Prospective Comparative Study

**DOI:** 10.2196/97990

**Published:** 2026-10-05

**Authors:** Huyen Thi Thu Nguyen, Lien Thi Tran, Hieu Thi Nguyen, Trang Thi Huyen Phan, The Anh Ngo

**Affiliations:** 1 Viet Tiep Hospital Hai Phong Vietnam; 2 Hai phong University Of Medicine and Pharmacy Hai Phong Vietnam

**Keywords:** dengue fever, N-acetylcysteine, marked aminotransferase elevation, liver enzymes, prospective study, Vietnam

## Abstract

**Background:**

Dengue fever can lead to significant hepatic involvement and marked aminotransferase elevation and is associated with increased morbidity and prolonged hospitalization. N-acetylcysteine (NAC) has been proposed as a supportive therapy for dengue-induced liver injury. However, prospective comparative evidence from endemic regions such as Vietnam remains limited.

**Objective:**

This study aimed to evaluate the association between intravenous NAC administration and clinical outcomes (primarily length of hospital stay), as well as the secondary effects of NAC on liver transaminase levels (posttreatment aspartate aminotransferase [AST] and alanine aminotransferase [ALT] and their changes from baseline) and platelet recovery in patients with dengue fever with marked aminotransferase elevation (AST or ALT ≥400 U/L).

**Methods:**

A prospective, nonrandomized comparative study was conducted at the Department of Tropical Diseases, Viet Tiep Hospital, Hai Phong, Vietnam, from June to December 2024. A total of 128 adult patients with laboratory-confirmed dengue fever and marked aminotransferase elevation (AST and/or ALT ≥400 U/L) were enrolled. Treatment allocation was not randomized and was determined by the treating physicians based on aminotransferase elevation and overall clinical condition. Patients received either intravenous NAC (100 mg per kilogram per day) plus standard supportive care (NAC group; n=68) or standard care alone (non-NAC group; n=60). The primary outcome was length of hospital stay. Secondary outcomes included liver transaminase levels and platelet recovery at discharge.

**Results:**

Baseline AST, ALT, bilirubin, and international normalized ratio values were broadly comparable between groups, although several clinical and hematological characteristics differed at baseline. The NAC group had a significantly shorter hospital stay (mean 5.01, SD 1.28 vs 6.12, SD 1.25 days; *P*<.001). The AST and ALT levels were significantly lower in the NAC group (AST: mean 154.83, SD 67.22 vs 184.62, SD 79.88 U/L and *P*=.02; ALT: mean 113.09, SD 33.31 vs 156.35, SD 73.66 U/L and *P*=.004). The posttreatment platelet count was significantly higher in the NAC group (median 31.50, IQR 19.00-50.15 G/L vs 17.00, IQR 8.75-36.23 G/L; *P*=.001). The median increase in platelet count was also greater in the NAC group (15.05, IQR 7.05-26.10 G/L vs 6.95, IQR 4.22-15.43 G/L; *P*=.001). No severe infusion-related adverse events were documented during the study period.

**Conclusions:**

In this prospective, nonrandomized comparative study, intravenous NAC administration was associated with shorter hospitalization and lower posttreatment aminotransferase levels in adult patients with dengue fever and marked aminotransferase elevation. No severe infusion-related adverse events were documented during the study period. However, the nonrandomized design and potential for confounding limit causal inference. Larger, multicenter randomized controlled trials are warranted to clarify the therapeutic role of NAC in dengue-related hepatic complications.

## Introduction

Dengue fever is a vector-borne viral infectious disease caused by dengue virus (DENV) and transmitted primarily by the *Aedes aegypti* and *Aedes albopictus* mosquitoes [1]. It represents a major global public health challenge, particularly in tropical and subtropical regions.

According to the World Health Organization, dengue cases have increased dramatically in recent decades. In 2023, over 5 million cases and more than 5000 deaths were reported across more than 80 countries and territories. Vietnam alone reported 149,557 cases, including 36 fatalities in that period [2].

Hepatic involvement is one of the most clinically significant complications of dengue fever. DENV-induced liver injury ranges from mild transaminase elevations to fulminant acute liver failure and is associated with higher mortality risk and prolonged hospitalization. Clinical manifestations of hepatic damage include nausea, vomiting, anorexia, abdominal pain, elevated transaminases, and hyperbilirubinemia [3,4].

The pathogenesis of DENV-induced liver damage remains incompletely understood. Proposed mechanisms include direct viral cytopathic effects, hypoxia-mediated injury, and immune-mediated hepatocyte apoptosis [3]. During acute infection, antioxidant enzyme levels, including glutathione peroxidase and glutathione reductase, decline, whereas aspartate aminotransferase (AST) and alanine aminotransferase (ALT) rise [5]. Experimental murine studies have demonstrated that DENV-induced acute liver failure involves proinflammatory cytokines (interleukin [IL]-2, IL-6, IL-8, and IL-22) and that N-acetylcysteine (NAC) attenuates liver damage, reduces hepatocyte apoptosis, and inhibits viral replication in HepG2 cells via interferon-mediated antiviral pathways [6]. NAC also preserved antioxidant enzyme levels and redox balance in DENV-infected mouse livers [7,8].

Supportive care and close clinical monitoring are central to dengue management [9]. NAC is an established antioxidant and glutathione precursor with well-characterized hepatoprotective properties [10,11]. It is recommended by the European Association for the Study of the Liver for acute liver failure from any cause [12] and has been used in dengue-associated acute liver injury in several Asian countries, with consistently positive outcomes reported [13-15]. However, robust randomized controlled trial evidence specifically on DENV-induced hepatic injury is lacking [16]. The 2023 Vietnamese Ministry of Health guidelines recommend NAC for patients with dengue fever with AST or ALT of 1000 U/L or higher [17]. The threshold of AST or ALT of at least 400 U/L was selected for this study to evaluate NAC at an earlier stage of marked aminotransferase elevation. This threshold should be regarded as an exploratory study criterion rather than an established indication for routine NAC treatment. The nonrandomized findings are intended to generate hypotheses and do not support changing clinical guidelines without confirmation in randomized controlled trials.

## Methods

### Study Design and Setting

This was a prospective, nonrandomized observational comparative study conducted at the Department of Tropical Diseases, Viet Tiep Hospital, Hai Phong, Vietnam, from June 1, 2024, to December 31, 2024. This study aimed to evaluate the association between NAC administration and clinical outcomes and the safety of intravenous NAC as an adjunctive therapy in adult patients with dengue fever complicated by marked aminotransferase elevation. No a priori sample size calculation was performed. This study used a prospective consecutive convenience sample comprising all eligible adult patients with dengue fever with marked aminotransferase elevation (AST or ALT ≥400 U/L) recruited during the predefined study period from June 2024 to December 2024 (N=128; n=68 in the NAC group and n=60 in the non-NAC group).

The detailed patient flow is shown in Figure 1.

**Figure 1:**
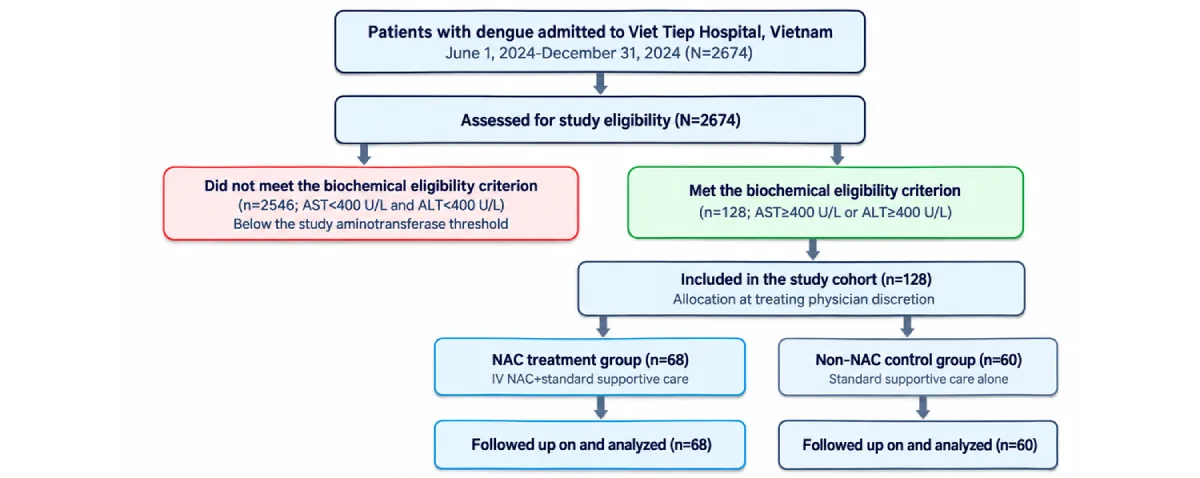
Flow diagram of patient screening, inclusion, and analysis. ALT: alanine aminotransferase; AST: aspartate aminotransferase; IV: intravenous; NAC: N-acetylcysteine.

### Participants

A total of 2674 patients with dengue fever were initially screened during the study period (June 1, 2024 to December 31, 2024). Of these, 2546 (95.2%) patients did not meet the biochemical threshold for marked aminotransferase elevation (AST or ALT<400 U/L). Among the 128 (4.8%) patients who met the inclusion criterion, no additional patients were excluded based on predefined exclusion criteria.

Ultimately, 128 patients were allocated to either the NAC group (n=68, 53.1%) or the non-NAC group (n=60, 46.9%) based on physician discretion. All 128 enrolled patients completed follow-up and were included in the final analysis.

Patients were eligible for inclusion if they met all the following criteria: (1) age of 18 years or above; (2) laboratory-confirmed dengue fever, defined as a positive dengue NS1 antigen and/or positive dengue immunoglobulin M antibody; and (3) marked aminotransferase elevation, defined as AST of 400 U/L or higher and/or ALT of 400 U/L or higher (the entry threshold of AST or ALT ≥400 U/L was selected as an exploratory study criterion to evaluate potential benefits of NAC at an earlier stage of marked aminotransferase elevation. This cutoff does not represent an established routine indication for NAC or a definition of acute liver failure, and its exploratory nature is recognized).

Patients were excluded if they met any of the following criteria: preexisting chronic liver disease (chronic hepatitis B or C, alcoholic liver disease, or cirrhosis), history of hepatotoxic drug use prior to admission (excluding standard therapeutic-dose paracetamol for symptomatic fever management), pregnancy or lactation, known hypersensitivity to NAC, or age under 18 years.

### Treatment Protocol

Treatment allocation was not randomized. Eligible patients were allocated to 2 groups based on clinical judgment in routine practice, reflecting real-world clinical decision-making. Intravenous NAC was initiated at the discretion of the treating physician after consideration of aminotransferase elevation and the patient’s overall clinical condition [17].

All included patients presented with marked aminotransferase elevation (AST or ALT ≥400 U/L) an exploratory threshold selected for this study. Clinicians tended to initiate intravenous NAC in patients presenting with overt hepatic involvement (eg, hepatomegaly and right upper quadrant tenderness) or rapidly progressive transaminitis accompanied by severe systemic symptoms (eg, persistent vomiting), reflecting a targeted pragmatic approach. Patients with stable transaminitis and milder clinical features were managed through standard supportive care alone [17].

In the NAC group, patients received intravenous NAC (BFS-Depara; Ha Noi CPC1 Pharmaceutical JSC) at 100 mg per kilogram per day diluted in 5% glucose solution and administered via slow intravenous infusion. NAC therapy was initiated at the discretion of the treating physician. Treatment was continued for 3 days, with earlier discontinuation at the discretion of the treating physician based on the patient’s clinical and biochemical response.

In the non-NAC (control) group, patients received standard supportive care for dengue fever, including fluid management, antipyretics, and close clinical monitoring, without NAC administration.

Standard supportive care was provided according to the Vietnamese national dengue management guideline and institutional practice [17]. It included clinically guided fluid management; symptomatic treatment where appropriate; serial assessment of vital signs and warning signs; complete blood count monitoring; liver biochemical testing; and clinical surveillance for bleeding, plasma leakage, hemodynamic deterioration, and other dengue-related complications. The supportive treatment was individualized; the intensity and components of supportive care were not identical between groups.

### Clinical and Laboratory Monitoring

#### Overview

All patients underwent daily monitoring of clinical symptoms and signs; hematological parameters (complete blood count); and biochemical parameters, including AST, ALT, total bilirubin, and international normalized ratio (when indicated). Liver function tests were recorded at baseline (at NAC initiation or hospital admission for the non-NAC group) and at day 3 of treatment or at hospital discharge, whichever occurred first. Patients were closely observed for adverse reactions to NAC infusion, including hypersensitivity reactions, nausea, vomiting, and anaphylaxis.

#### Safety Assessment

Safety outcomes and infusion-related reactions were actively monitored through continuous clinical surveillance during intravenous NAC administration. Monitoring focused on hypersensitivity and anaphylactoid symptoms, including rash, pruritus, respiratory distress, hemodynamic changes, and gastrointestinal intolerance.

### Outcomes

The primary outcome was length of hospital stay (days). Secondary outcomes were changes in serum AST and ALT levels after treatment; platelet count recovery, assessed using the minimum platelet count at the end of the treatment assessment period (day 3 or hospital discharge, whichever occurred first); and change from baseline to this posttreatment measurement. In addition, the change in AST and ALT levels (Δ) was calculated.

### Statistical Analysis

Statistical analysis was performed using the SPSS software (IBM Corp). Continuous variables are expressed as means and SDs or medians and IQRs as appropriate. Categorical variables are presented as frequencies and percentages. Continuous variables were compared using the Student 2-tailed *t* test for normally distributed data or the Mann-Whitney *U* test for nonnormally distributed data. Categorical variables were compared using the chi-square or Fisher exact test.

### Ethical Considerations

The study protocol was approved by the scientific council and ethics committee of Viet Tiep Hospital, Hai Phong, Vietnam. Written informed consent was obtained from all participants or their legal guardians prior to enrollment. All procedures were conducted in accordance with the principles of the Declaration of Helsinki.

## Results

### Patient Characteristics

During the study period, 128 patients with laboratory-confirmed dengue fever and marked aminotransferase elevation (AST and/or ALT ≥400 U/L) met the inclusion criteria. Of these, 68 received intravenous NAC, and 60 received standard supportive care alone.

Baseline demographic characteristics are summarized in Table 1. The mean age was 47.79 (SD 17.43) years in the NAC group and 52.81 (SD 14.11) years in the non-NAC group. Although the mean age was numerically lower in the NAC group, the between-group difference was not statistically significant (*P*=.07). Sex distribution was comparable between groups, with no statistically significant differences.

**Table 1:** Demographic characteristics of study patients (N=128).

Characteristics			NAC^a^ (n=68)		Non-NAC (n=60)		Total
**Sex, n (%)**							
	Male	25 (36.8)		28 (46.7)		53 (41.4)	
	Female	43 (63.2)		32 (53.3)		75 (58.6)	
Age (y), mean (SD)			47.79 (17.43)		52.81 (14.11)		50.14 (16.10)

^a^NAC: N-acetylcysteine.

### Baseline Clinical and Laboratory Features

Baseline clinical and paraclinical characteristics are shown in Table 2. All 128 included patients had concurrent elevations in both AST and ALT, and no patient had isolated AST elevation. The NAC group had a longer duration of fever prior to admission (mean 4.57, SD 1.31 vs 3.92, SD 1.41 days; *P*=.007) and higher rates of vomiting (37/68, 54.4% vs 19/60, 31.7%; *P*=.01) and hepatomegaly (37/68, 54.4% vs 12/60, 20%; *P*<.001). The higher prevalence of mucosal bleeding in the non-NAC group represented a baseline imbalance. Its cause could not be determined from the available data, and residual confounding related to bleeding severity may remain. Baseline laboratory parameters, including AST, ALT, total bilirubin, hemoglobin, hematocrit, platelet nadir, white blood cell count, and international normalized ratio, were not significantly different between groups (*P*>.05 for all comparisons), indicating comparable baseline biochemical severity despite more pronounced clinical manifestations in the NAC group.

**Table 2:** Baseline clinical and paraclinical characteristics.

Characteristics	NAC^a^ (n=68)	Non-NAC (n=60)	*P* value
Fever duration (d), mean (SD)	4.57 (1.31)	3.92 (1.41)	.007
Right upper quadrant pain, n (%)	29 (42.6)	13 (21.7)	.01
Nausea, n (%)	34 (50)	25 (41.7)	.35
Vomiting, n (%)	37 (54.4)	19 (31.7)	.01
Hemorrhage, n (%)	28 (41.2)	35 (58.3)	.053
Mucosal bleeding, n (%)	17 (25)	30 (50)	.003
Pleural effusion, n (%)	33 (48.5)	21 (35)	.12
Gallbladder wall thickening, n (%)	36 (52.9)	24 (40)	.14
Hepatomegaly, n (%)	37 (54.4)	12 (20)	<.001
Dengue severity—grade 2, n (%)	53 (77.9)	54 (90)	.59
Dengue severity—grade 3, n (%)	5 (7.4)	6 (10)	.59
Hemoglobin (g/L), mean (SD)	163.77 (15.97)	142.55 (12.05)	.07
Maximum hematocrit (L/L), mean (SD)	0.44 (0.06)	0.44 (0.06)	.06
Minimum platelet count (G/L), median (IQR)	12,40 (5.95-26.17)	6.00 (2.65-12.38)	.003
White blood cell count (G/L), mean (SD)	4.95 (3.55)	3.67 (2.30)	.55
INR^b^, mean (SD)	1.01 (0.08)	1.02 (0.09)	.27
AST^c^ (U/L), mean (SD)	609.68 (275.10)	511.53 (159.50)	.09
ALT^d^ (U/L), mean (SD)	425.12 (156.13)	419.96 (162.93)	.80
Total bilirubin (μmol/L), mean (SD)	12.45 (4.75)	13.65 (5.24)	.19

^a^NAC: N-acetylcysteine.

^b^INR: international normalized ratio.

^c^AST: aspartate aminotransferase.

^d^ALT: alanine aminotransferase.

### Treatment Outcomes

#### Overview

Posttreatment outcomes are summarized in Table 3.

**Table 3:** Treatment outcomes at discharge.

Outcomes	NAC^a^ (n=68)	Non-NAC (n=60)	*P* value
Hospital stay (d), mean (SD)	5.01 (1.28)	6.12 (1.25)	<.001
Posttreatment AST^b^ (U/L), mean (SD)	154.83 (67.22)	184.62 (79.88)	.02
Posttreatment ALT^c^ (U/L), mean (SD)	113.09 (33.31)	156.35 (73.66)	.004
ΔAST (U/L), mean (SD)	454.85 (261.85)	326.91 (170.44)	.002
ΔALT (U/L), mean (SD)	312.03 (154.21)	263.62 (153.99)	.08
Posttreatment platelet count (G/L), median (IQR)	31.50 (19.00-50.15)	17.00 (8.75-36.23)	.001
Change in platelet count (G/L), median (IQR)	15.05 (7.05-26.10)	6.95 (4.22-15.43)	.001

^a^NAC: N-acetylcysteine.

^b^AST: aspartate aminotransferase.

^c^ALT: alanine aminotransferase.

#### Length of Hospital Stay

The mean duration of hospitalization was significantly shorter in the NAC group than in the non-NAC group (5.01, SD 1.28 vs 6.12, SD 1.25 days; *P*<.001).

#### Liver Enzyme Recovery

Mean posttreatment AST levels were significantly lower in the NAC group (154.83, SD 67.22 vs 184.62, SD 79.88 U/L; *P*=.02), as were posttreatment mean ALT levels (113.09, SD 33.31 vs 156.35, SD 73.66 U/L; *P*=.004). The mean change in AST was 454.85 (SD 261.85) U/L in the NAC group vs 326.91 (SD 170.44) U/L in the non-NAC group (*P*=.002). The mean change in ALT was 312.03 (SD 154.21) vs 263.62 (SD 153.99), respectively (*P*=.08).

#### Platelet Recovery

The posttreatment platelet count was significantly higher in the NAC group (median 31.50, IQR 19.00-50.15 G/L vs 17.00, IQR 8.75-36.23 G/L; *P*=.001). The median increase in platelet count was also greater in the NAC group (15.05, IQR 7.05-26.10 G/L vs 6.95, IQR 4.22-15.43 G/L; *P*=.001). However, because platelet counts were not assessed at uniform predefined time points for all participants, the study could not determine whether NAC accelerated the rate of platelet recovery.

#### Safety

All patients recovered and were discharged in stable condition. No patients required intensive care unit admission, and no severe infusion-related adverse events were recorded during the study period.

## Discussion

### Principal Findings

In this prospective, nonrandomized comparative study of 128 adult patients with dengue fever and marked aminotransferase elevation, intravenous NAC administration was associated with a shorter duration of hospitalization and lower posttreatment AST and ALT levels compared with standard supportive care alone [17]. The mean hospital stay was approximately 1 day shorter in the NAC group (5.01, SD 1.28 vs 6.12, SD 1.25 days; *P*<.001). Posttreatment AST and ALT levels were also significantly lower in the NAC group. These associations were observed despite the NAC group presenting with more pronounced clinical manifestations at baseline, including longer fever duration and higher rates of vomiting and hepatomegaly. NAC was well tolerated, with no infusion-related adverse events documented.

### Detailed Discussion

The observed association between NAC administration and shorter hospital stay is consistent with prior reports, including the case series by Dissanayake et al [15], that have demonstrated shorter hospitalization among patients with dengue fever with severe hepatitis treated with NAC. A reduction of approximately 1 hospital day may be operationally relevant during dengue outbreaks, particularly in resource-constrained settings. However, the clinical and economic importance of this difference was not directly evaluated and should be confirmed in prospective randomized and health economic studies.

The more pronounced reduction in AST and ALT levels in NAC-treated patients aligns with the known antioxidant and hepatoprotective mechanisms of NAC. DENV infection is associated with oxidative stress, mitochondrial dysfunction, and immune-mediated hepatocyte injury [5,6]. As a glutathione precursor, NAC may mitigate oxidative damage and promote hepatocellular recovery, thereby accelerating the normalization of liver enzymes. Experimental data have also suggested that NAC may exert direct antiviral effects by inhibiting DENV replication via interferon-mediated pathways [6], although viral load was not assessed in the present study.

Alternative explanations for the observed findings must be considered. The NAC group had a longer duration of fever prior to admission (mean 4.57, SD 1.31 vs 3.92, SD 1.41 days; *P*=.007). Patients presenting later in the course of dengue may have been closer to the spontaneous recovery phase; therefore, part of the observed biochemical improvement may reflect the natural disease course rather than NAC administration alone. The higher prevalence of vomiting and hepatomegaly in the NAC group may reflect physician selection of patients with more prominent hepatic manifestations. However, the present study cannot determine whether hepatomegaly modifies the association between NAC and clinical outcomes. This question requires a prespecified subgroup analysis within a randomized trial.

Furthermore, the coexistence of hepatomegaly and marked aminotransferase elevation may represent a more clinically pronounced pattern of dengue-associated hepatic involvement. The potential biological effects of NAC, including restoration of glutathione availability and attenuation of oxidative stress, provide a rationale for further evaluating whether patients with this combined clinical and biochemical phenotype might derive greater benefit from NAC. However, the present observational study was not designed to determine whether hepatomegaly modifies the association between NAC treatment and clinical outcomes. This observation should therefore be regarded as hypothesis generating. Future prospective studies with prespecified subgroup and interaction analyses are warranted to determine whether this phenotype identifies patients who may derive greater benefit from NAC.

Although both groups received care according to the same institutional dengue management framework, the intensity and components of supportive treatment were not quantified in sufficient detail. Differences in fluid management, antiemetic treatment, oral intake, or other supportive interventions may have contributed to the observed outcomes.

Mucosal bleeding was assessed at baseline (upon admission), before treatment initiation. Therefore, the higher prevalence in the non-NAC group (30/60, 50% vs 17/68, 25%) represents a baseline imbalance rather than a treatment effect. As this was a nonrandomized study, the specific reason for this imbalance could not be determined from the available data, and residual confounding related to differences in disease or bleeding severity cannot be excluded.

An interesting finding was the difference in platelet counts between groups. While NAC is not conventionally regarded as a thrombopoietic agent, this effect may be indirect, reflecting reduced systemic inflammation and oxidative stress rather than a direct pharmacological action on platelet production. Hepatic recovery could theoretically influence platelet production through restoration of thrombopoietin synthesis; however, dengue-related thrombocytopenia is multifactorial and may also involve bone marrow suppression, immune-mediated platelet destruction, peripheral consumption, and systemic inflammation. Because thrombopoietin was not measured and platelet counts were not assessed at fully standardized time points, this study cannot establish a mechanistic link between NAC, hepatic recovery, thrombopoietin production, and platelet changes. Given the observational nature of this study, this association warrants cautious interpretation.

NAC was well tolerated in all patients, with no infusion-related adverse events documented, consistent with its established safety profile in paracetamol-induced and non–paracetamol-related acute liver failure [12,18].

The findings may be most relevant to adult patients treated in comparable dengue-endemic hospital settings with access to serial aminotransferase testing and safe intravenous infusion monitoring. However, generalizability is limited by the single-center design, local treatment practices, physician-directed treatment allocation, and selected study population. The results should not yet be interpreted as supporting the routine use of NAC at an aminotransferase threshold of 400 U/L in other health care systems.

To our knowledge, this is among the first prospective comparative studies evaluating intravenous NAC specifically for dengue-associated marked aminotransferase elevation in Hai Phong, Vietnam, contributing to the limited evidence base in this field.

### Limitations

This study has several important limitations.

First, the single-center, nonrandomized design with physician-directed treatment allocation may have introduced selection bias and confounding by indication. No formal randomization sequence or allocation concealment procedure was used, and blinding was not possible.

Second, the sample size was modest, and no a priori sample size calculation was performed; the study used a prospective convenience sample of eligible patients during the predefined study period, which may limit the precision and generalizability of the findings.

Third, several baseline clinical characteristics differed between groups, including fever duration, vomiting, hepatomegaly, and mucosal bleeding. These imbalances, together with differences in supportive treatment and incompletely measured prehospital paracetamol exposure, may have contributed to the observed outcomes, and residual or unmeasured confounding cannot be excluded.

Fourth, supportive treatment was individualized according to clinical condition, and its intensity and specific components were not quantified in sufficient detail. Differences in fluid management, antiemetic treatment, oral intake, or other supportive interventions may therefore have influenced length of hospital stay and biochemical outcomes.

Fifth, platelet counts were not assessed at uniform predefined time points for all participants; consequently, this study could not adequately compare platelet trajectories or determine whether NAC accelerated platelet recovery.

Sixth, secondary biochemical markers, including alkaline phosphatase, gamma-glutamyl transferase, serum albumin, and creatinine, were not systematically measured at uniform time points, limiting a more comprehensive longitudinal assessment of hepatic and renal involvement.

Seventh, viral load and dengue serotype were not measured, precluding assessment of their potential influence on treatment response and clinical outcomes.

Eighth, safety monitoring relied on routine inpatient clinical surveillance rather than a standardized prospective adverse event grading system. Although no severe infusion-related adverse events were documented, mild or transient reactions may have been underrecorded.

Finally, follow-up was limited to the hospitalization period, and longer-term outcomes were not assessed.

### Conclusions

In this prospective, nonrandomized comparative study, intravenous NAC administration was associated with shorter hospitalization and lower posttreatment aminotransferase levels in adult patients with dengue fever and marked aminotransferase elevation. No severe infusion-related adverse events were documented during the study period, although routine clinical surveillance may have underestimated mild or transient reactions. However, the nonrandomized design, potential for physician treatment bias, baseline clinical imbalances, and unmeasured confounding limit causal inference. These findings suggest that NAC may have potential as an adjunctive therapy in this clinical setting. Larger, multicenter randomized controlled trials with standardized supportive care protocols and prespecified end points are warranted to clarify the therapeutic role of NAC in dengue-related hepatic complications and determine whether the observed associations reflect a true treatment effect.

## Abbreviations

ALT: alanine aminotransferase
AST: aspartate aminotransferase
DENV: dengue virus
IL: interleukin
NAC: N-acetylcysteine

## Data Availability

The datasets supporting the conclusions of this study are available from the corresponding author (HN) on reasonable request.
